# Clinical course of *Capnocytophaga canimorsus* bacteremia from acute onset to life crisis

**DOI:** 10.1002/ccr3.3511

**Published:** 2020-11-11

**Authors:** Hideki Tsunoda, Hidetomo Nomi, Kunihiko Okada, Tsuneaki Kenzaka

**Affiliations:** ^1^ Department of Family Medicine Shiga University of Medical Science Otsu Japan; ^2^ Department of Cardiology Saku Central Hospital Advanced Care Center Saku Japan; ^3^ Department of Emergency Medicine Saku Central Hospital Advanced Care Center Saku Japan; ^4^ Department of Internal Medicine Hyogo Prefectural Tamba Medical Center Tamba Japan; ^5^ Division of Community Medicine and Career Development Kobe University Graduate School of Medicine Kobe Japan

**Keywords:** animal bite, *Capnocytophaga canimorsus* bacteremia, fulminant sepsis

## Abstract

*Capnocytophaga canimorsus* bacteremia can present without signs of sepsis just after onset. For patients with fever, discerning the history of an animal bite is crucial. If it is positive for a dog bite, antibiotic treatment should be started.

## Graphical abstract

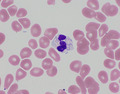

## 1 INTRODUCTION

*Capnocytophaga canimorsus* bacteremia can present as fever and headache without signs of severe sepsis and can be misdiagnosed as a viral infection. For patients with fever whose source is unclear, the history of an animal bite should be taken and considered for the possibility of potentially fatal *Capnocytophaga canimorsus* bacteremia.

*Capnocytophaga canimorsus* is an anaerobic gram‐negative bacterium first described by Bobo et al in 1976.^1^ It is found in the oral cavity of dogs and cats. Infection with this bacterium after a dog bite can result in bacteremia, meningitis, and infective endocarditis. Moreover, some cases of bacteremia can result in fulminant sepsis, with a high mortality rate of 26%.^2^ As *C canimorsus* is usually susceptible to penicillin,^2^ amoxicillin/clavulanic acid is recommended as a prophylactic treatment after a dog bite.^3^ Despite its high virulence, *C canimorsus* is a slow‐growing bacterium and its isolation from blood cultures takes time.^4^ Therefore, the diagnosis and treatment of *C canimorsus* bacteremia are sometimes challenging, unless the history of a dog bite is clear.^5^ When patients present to a hospital, they usually show signs of shock or multiorgan failure. However, a case without any life‐threatening signs at the initial presentation is rare. We report here the case of *C canimorsus* bacteremia that presented only with acute fever and headache immediately after the onset without any abnormality in vital signs or laboratory tests except for mild elevation of (C‐reactive protein) CRP.

## 2 CASE REPORT

A 67‐year‐old Japanese man presented to the emergency department with fever and a constant headache that began 5 and 9 hours ago, respectively. The location of the headache was at the back of his head. He described the pain as pulsating and rated it 10 on a 0 to 10‐point Numerical Rating Scale. He had no gastrointestinal (GI) symptoms such as nausea, vomiting, or diarrhea. He had a past medical history of hyperlipidemia and type 2 diabetes that was well controlled by sitagliptin alone; his hemoglobin A1c level was 6.1%. He drank 2‐3 glasses of Shochu (alcoholic beverage) every day; however, his previous liver function tests were normal. Therefore, we considered him to be immunocompetent.

His blood pressure, pulse rate, respiratory rate, and body temperature were 123/78 mm Hg, 110/min, 18/min, and 39.1°C, respectively. He was alert and oriented. Physical examination did not reveal any abnormalities. Meningeal irritation symptoms were negative. We performed blood tests, urine analysis, chest X‐ray, computed tomography (CT) scan of the head, and lumbar puncture to find the source of the fever and rule out meningitis. Laboratory tests revealed normal findings (white blood cell count, 8.3 × 10^9^/L; hemoglobin, 144 g/L; platelets, 169 × 10^9^/L; creatinine, 0.65 mg/dL; blood glucose, 117 mg/dL), although mild elevation of CRP levels was observed (2.5 mg/dL: normal range 0.0‐0.3 mg/dL). Lumbar puncture and other tests were also normal (appearance, clear; no xanthochromy; cell counts 1/µL [monocyte]; glucose, 80 mg/dL). Although the diagnosis and the source of fever were unclear, he was admitted to our hospital for observation for headache. Blood culture was done. We treated him with drip infusion and did not start antibiotics as our initial diagnosis was a viral infection and there were no signs of a severe bacterial infection. We planned to start antibiotics if the results of the blood culture came back positive.

Six hours after hospital admission, he began having frequent vomiting and diarrhea episodes. We continued to observe him as his vital signs remained normal. Eleven hours later, he became agitated and his consciousness deteriorated. His oxygen saturation level decreased and petechia appeared on his upper and lower limbs. He was re‐evaluated with blood tests and contrast‐enhanced CT scan for chest, abdomen, and pelvis. His blood tests showed signs of disseminated intravascular coagulopathy (DIC) and multiorgan failure (MOF): pancytopenia (white blood cell count, 0.6 × 10^9^/L; hemoglobin, 97 g/L; platelets, 12 × 10^9^/L), renal dysfunction (BUN, 25 mg/dL; creatinine, 2.52 mg/dL), elevated aminotransferase (AST, 386 IU/L; ALT, 90 IU/L), and coagulopathy (PT‐INR, 3.15; D‐dimer, 55.4 mcg/mL; APTT > 180 seconds). The CT failed to demonstrate any possible source of infection and his spleen appeared to be normal with no signs of hypoplasia. Even though the origin was unclear, fulminant sepsis was apparent; we transferred the patient to a tertiary hospital nearby for ICU care. At this time, further history taking revealed that the patient had been bitten by his dog 7 days before admission, although we were unable to find any wounds or scar even after careful physical re‐examination after learning of this history. Subsequently, we considered severe infection due to an animal bite, such as *C canimorsus* or *Pasteurella multocida* infection, as the differential diagnosis.

**FIGURE 1:**
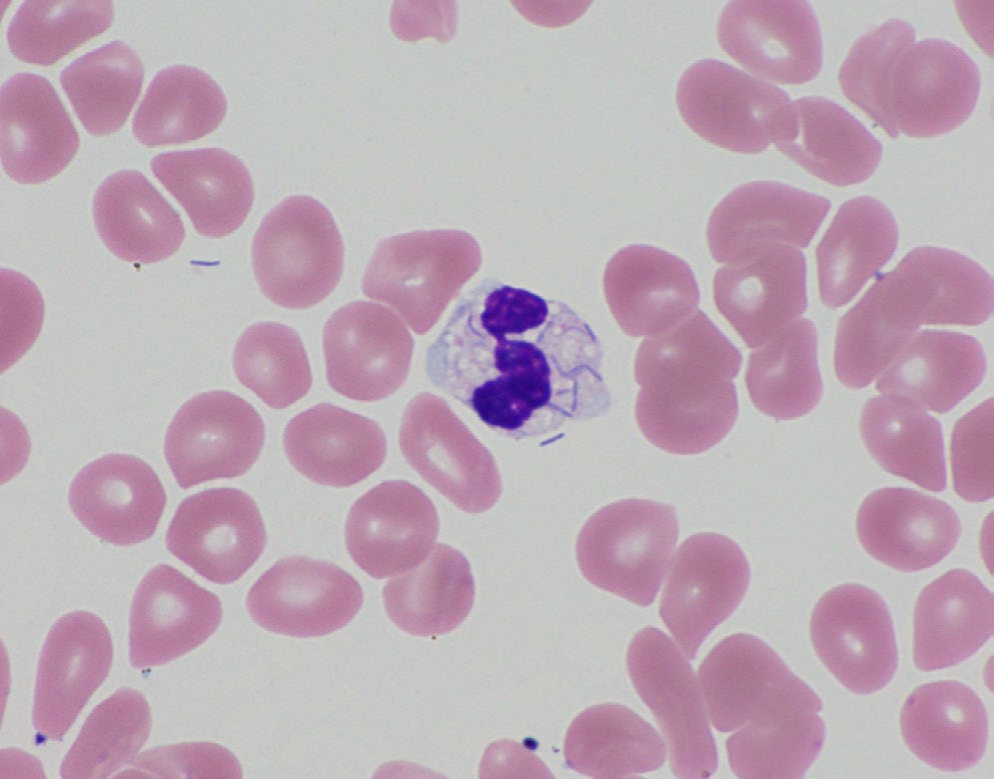
Blood smear after ICU admission that shows bacteria in a leukocyte

After transfer, he was admitted to the ICU. He became hypotensive and was given a bolus of normal saline and started on noradrenaline. He was also given broad‐spectrum antibiotics (levofloxacin 500 mg and meropenem 1 g). He was intubated due to respiratory failure. Just after admission, we received a report that his blood smear (Giemsa staining) showed the presence of bacteria in leukocytes (Figure 1). In spite of intensive care, he remained hypotensive and suffered from complications of GI bleeding. After 41 hours of presentation, the patient died as a result of DIC and MOF. The day following the patient's death, blood cultures tested positive for bacteremia; 28 days after his death, the bacteria were identified as *C canimorsus* using 16S rRNA gene sequence analysis.

## 3 DISCUSSION

We present an extremely rare case of fatal *C canimorsus* bacteremia that did not show characteristic symptoms at the early stage just after onset. Thus, it was difficult to make an early diagnosis at his emergency visit. Careful history taking enabled us to consider a possible diagnosis of *C canimorsus* bacteremia before blood cultures came back positive, although this was only after the patient's condition had deteriorated.

*Capnocytophaga canimorsus* bacteremia causes fulminant sepsis and presents with severe abnormalities in vital signs and laboratory tests; hence, the diagnosis of sepsis is usually not difficult. However, we observed that when a patient presents to the hospital at a very early stage just after onset, laboratory tests may not be helpful in the diagnosis of severe sepsis. This may result in the misdiagnosis of this condition as a viral infection. Other studies have reported similar cases; however, in these cases, a dog bite was visible on presentation, unlike in the present case. Hence, in those cases, the physicians were able to diagnose a specific bacteremia after an animal bite, such as *C canimorsus* or *Pasteurella multocida*.^6^, ^7^ Furthermore, the initial symptoms of *C canimorsus* bacteremia present more often as GI symptoms such as vomiting (31%), diarrhea (26%), or mental confusion (23%) than as unspecific symptoms such as headache (18%)^8^; this initial presentation made the diagnosis of *C canimorsus* bacteremia difficult in this case.

For patients with fever whose source is unclear, clinicians need to take a careful history of animal bites and, if it is positive, they must not hesitate to start antibiotics even with a short‐lived fever just and no clinical signs of sepsis. Further, history taking in such cases is crucial because most patients have no signs of infection at the wound site^2^ and patients do not readily disclose the history of an animal bite without context or prompting. According to the best practices for sepsis treatment, identification of this type of infection and administration of antibiotics as early as possible is vital.

In conclusion, when a patient presents to the hospital just after onset, *C canimorsus* bacteremia can present without abnormalities in vital signs or laboratory tests that are suggestive of sepsis. For a patient with a fever whose source is unclear, careful history taking of animal bite is important and if found positive, one should not hesitate to start antibiotics.

## CONFLICT OF INTEREST

The authors declare no conflicts of interest associated with this manuscript.

## AUTHOR CONTRIBUTIONS

HT: managed the case and redaction and corrected the manuscript. HN and KO: managed the case and assisted with redaction, correction, and reconstruction of the manuscript. TK: assisted with redaction, correction, and reconstruction of the manuscript. All authors read and approved the final manuscript.

## ETHICS APPROVAL AND CONSENT TO PARTICIPATE

Not applicable.

## Data Availability

All data generated or analyzed during this study are included in this published article.
